# Microencapsulated docosahexaenoic acid increases the Omega-3 Index and attenuates the physiological impact of eccentric exercise in physically trained adults: a 12-week double-blind placebo-controlled trial

**DOI:** 10.1007/s00394-026-03998-6

**Published:** 2026-05-29

**Authors:** Ryan Anthony, Michael J. Macartney, Peter L. McLennan, Amal Elhage, Ronald Sluyter, John A. Sampson, Todd W. Mitchell, Gregory E. Peoples

**Affiliations:** 1 Graduate School of Medicine, Faculty of Science Medicine and Health, University of Wollongong, Wollongong, NSW 2522 Australia; 2 Health Innovations, University of Wollongong, Wollongong, Australia; 3 Molecular Horizons, University of Wollongong, Wollongong, NSW Australia; 4 School of Science, University of Wollongong, Wollongong, NSW Australia; 5 School of Medical, Indigenous and Health Sciences, University of Wollongong, Wollongong, NSW Australia

**Keywords:** Omega-3 Index, DHA, Eccentric exercise, Muscle damage, Inflammation, Physically trained adults

## Abstract

**Purpose:**

This study investigated the effects of docosahexaenoic acid (DHA) supplementation on delayed onset muscle soreness (DOMS), physical function, and inflammation following eccentric exercise-induced muscle damage in physically trained male and female adults (training ≥ 5d/wk).

**Methods:**

Thirty-eight participants (12 Control, 26 DHA) completed a 12-week double-blind, placebo-controlled matched-pair trial. The Control group received high-oleic acid tablets. The DHA group received 715 mg/d of microencapsulated DHA tablets. Participants performed eccentric cycling at weeks 0 and 12, with assessments conducted pre-exercise, 0-h, 24-h, and 48-h post-exercise. The primary outcomes were DOMS (visual analogue scale) and the Omega-3 Index (O3I) (estimated by finger-stick dry blood spot). Secondary outcomes included neuromuscular function and inflammatory cytokines.

**Results:**

O3I was not different between groups at week 0 but was elevated in the DHA group (∆2.43%, [95% CI; 2.10, 2.77], *P* < 0.001) and between Control at week 12 (*P* < 0.001). DOMS was lower at 24-h and 48-h post-exercise in the DHA group and between Control at week 12 (*P* < 0.01). At week 12, jump height and peak vertical force improved at 48-h post-exercise in the DHA group (*P* < 0.05), resulting in moderate (*d* = 0.60) and small (*d* = 0.32) effect sizes for 48-h post-exercise area under the curve (AUC) between groups, respectively. There were interaction effects for 48-h post-exercise AUC of IL-6 (*P* = 0.049), TNF-α (*P* = 0.010), and IL-10 (*P* = 0.026).

**Conclusion:**

A dietary achievable dose of DHA elevated the O3I and reduced DOMS in physically trained adults. These outcomes support protracted intake of DHA to attenuate the physiological impact of eccentric exercise and promote recovery.

**Supplementary Information:**

The online version contains supplementary material available at 10.1007/s00394-026-03998-6.

## Introduction

Eccentric exercise, characterised by muscle contracting while lengthening under tension, results in a temporary decrease in achievable muscle force and power and is often accompanied by perceived muscle soreness, and generation of inflammatory and muscle damage markers [1, 2]. The magnitude of these physiological disruptions are influenced by the intensity, modality and familiarity with the eccentric exercise task, whereby eccentric contractions performed at higher intensity (% of peak effort) [3], at longer muscle length [4], and faster contraction velocity [5] result in a more pronounced effect, especially with novel exercise tasks [6].

Delayed onset muscle soreness (DOMS) emerges after eccentric exercise, typically peaking 24-h to 48-h post-exercise, whereas peak levels of inflammatory and muscle damage markers are more variable [2]. The combined effects of direct and indirect mechanisms of muscle damage and inflammation after eccentric exercise impair physical performance and training consistency [7]. Hody et al. [8] classified the damage phases as initial mechanical damage involving calcium influx [9], and secondary damage, primarily involving the pro-inflammatory response, followed by an anti-inflammatory/pro-resolving phase [8]. While DOMS and inflammation are part of normal adaptation to muscle damaging exercise, excessive or unresolved inflammation can hinder muscle repair by promoting fibrosis instead of regeneration, resulting in prolonged soreness, decreased function, and delayed recovery, limiting subsequent athletic performance [10]. Understanding how to modulate the inflammatory response to balance effective muscle repair while minimising prolonged damage and soreness is crucial for optimising recovery and performance [7]. This has led to an intense focus in nutritional interventions centred around long chain omega-3 polyunsaturated fatty acid (LC n-3 PUFA) supplementation, which may mitigate excessive inflammation and improve muscle recovery [11].

LC n-3 PUFA, specifically eicosapentaenoic acid (EPA; 20:5*n*-3) and docosahexaenoic acid (DHA; 22:6*n*-3), uniquely modulate inflammation [12]. Most evident in chronic disease conditions where inflammation is often excessive or uncontrolled, EPA and DHA provide both anti-inflammatory and inflammation resolution properties [13]. These effects are mediated in part through altered eicosanoid production and their influence on intracellular signalling of pro-inflammatory cytokines [13]. More recently, these fatty acids have emerged as potential contributors to post-exercise recovery, including the mitigation of DOMS, restoration of muscle force and resolution of inflammation [14, 15]. Dietary consumption of preformed marine sourced EPA and/or DHA leads to their incorporation within cardiac [16] and skeletal muscle membranes, with the extent of incorporation varying according to muscle fibre type [17, 18]. Notably, DHA is preferentially incorporated into the phospholipid membranes of fast oxidative–glycolytic (type IIA) muscle fibres in animals deprived of dietary omega-3 fatty acids, with incorporation further augmented when omega-3 is supplied in the diet [18]. Of relevance, the preferential damage to type II muscle fibres following eccentric exercise is well established [19, 20], highlighting a potential specific role for DHA in modulating recovery from muscle damaging exercise.

Incorporation of EPA and DHA into cell membranes partially occurs at the expense of omega-6 PUFA, particularly arachidonic acid (AA; 20:4*n*-6) [16, 18]. DHA is the primary LC n-3 PUFA that replaces AA in skeletal muscle membranes [17, 18, 21]. This alteration in membrane fatty acid composition, whether by the incorporation of DHA, or EPA, and in this case, predominantly influenced by DHA, leads to reduced AA availability and a subsequent potential decrease in pro-inflammatory AA-derived prostaglandin E_2_ [22]. Supplementation with DHA has demonstrated potential in accelerating post-exercise recovery including attenuating strength loss [14], reducing DOMS through its antinociceptive properties [23] and its ability to lower cytokine levels following eccentric exercise [24]. Additionally, specialised pro-resolving mediators derived from EPA and DHA have been shown to effectively resolve acute inflammation [25]. Typical, over the counter non-steroidal anti-inflammatory drugs (NSAIDs), are often used by athletes [26], primarily acting to blunt the inflammatory response through inhibition of cyclooxygenase (COX). In contrast, specialised pro-resolving mediators, which include resolvins, protectins, and maresins, actively promote resolution of inflammation, accelerating tissue repair without impairing physiological adaptations [25]. The biological effects of these specialised pro-resolving mediators broadly include promoting the resolution of acute inflammation, exerting general anti-inflammatory effects, and potentially alleviating inflammation-associated pain related to DOMS [27–29]. More specifically, DHA-derived pro-resolving mediators have demonstrated pronounced analgesic properties in rodent models [30], particularly in the cellular pathways related to muscle lengthening under contraction [31].

Despite the promising effects of EPA and DHA in reducing inflammation and aiding muscle recovery following eccentric exercise, research findings remain mixed, primarily due to substantial study heterogeneity and low quality study designs as previously highlighted [11]. Key concerns include the lack of hypotheses for omega-3 mechanisms in recovery, resulting in arbitrary selection of EPA + DHA composition, dose, and duration [11]. Few studies measure whole blood levels of EPA + DHA, and even fewer assess biomarkers reflecting tissue levels, such as the Omega-3 Index (O3I; %EPA + DHA of total erythrocyte membrane fatty acids). Despite at least 10 further studies being published since study design concerns were raised [11], the same limitations persist, with only one study measuring erythrocyte EPA + DHA to confirm baseline status and ensure group separation [14]. Dietary achievable doses of LC n-3 PUFA over the long term, equivalent to approximately 2–3 fatty fish meals per week, can substantially increase the O3I in physically trained adults, enabling clear group separation [32]. However, time-course data suggest a minimum supplementation duration of eight weeks is required [32]. Additionally, no study has yet evaluated outcomes at both pre- and post-supplement periods, thereby preventing within-group analyses.

The aim of this study was to investigate the effects of a dietary achievable dose of DHA consumed by physically trained adults on the primary outcomes of the O3I over 12 weeks and DOMS in response to eccentric exercise-induced muscle damage. Secondary outcomes included muscle function and markers of inflammation during the 48-h recovery. It was hypothesised that an increased O3I, reflecting membrane incorporation of DHA, would improve physiological recovery following eccentric exercise-induced muscle damage, primarily demonstrated through a reduction in DOMS.

## Methods

### Participants

Physically trained adults (18–45 years), consuming ≤ 1 fatty fish meal per week, not consuming LC n-3 PUFA supplements and not taking anti-inflammatory medication were recruited from local sport and recreation facilities in the Illawarra region, NSW, Australia. A total of n = 12 Control group (33% female) and n = 26 DHA group (38% female) participants were recruited and completed the study (Supplementary Fig. 1). This study was approved by the University of Wollongong Human Research Ethics Committee (approval number: 2022/207) and was conducted in accordance with the Declaration of Helsinki. Full details on eligibility criteria, participant recruitment and selection can be found in Anthony et al. [32].

### Study design

Using a double-blind, placebo-controlled pair-matched design, this study investigated the effects of a dietary achievable dose of DHA microencapsulated in tablets consumed daily for 12-weeks, on DOMS, neuromuscular function and the inflammatory response during the 48-h recovery period following eccentric cycling-induced muscle damaging exercise. To ensure baseline consistency and equal workload during the eccentric cycling protocol, participants were pair-matched based on (i) estimated O3I, (ii) concentric peak power output, and (iii) 24-h muscle soreness prior to receiving group allocation. Tablet allocation was conducted by an independent researcher not involved in data collection or analysis, and all unmarked tablets were concealed in colour-coded opaque boxes to maintain blinding.

**Fig. 1:**
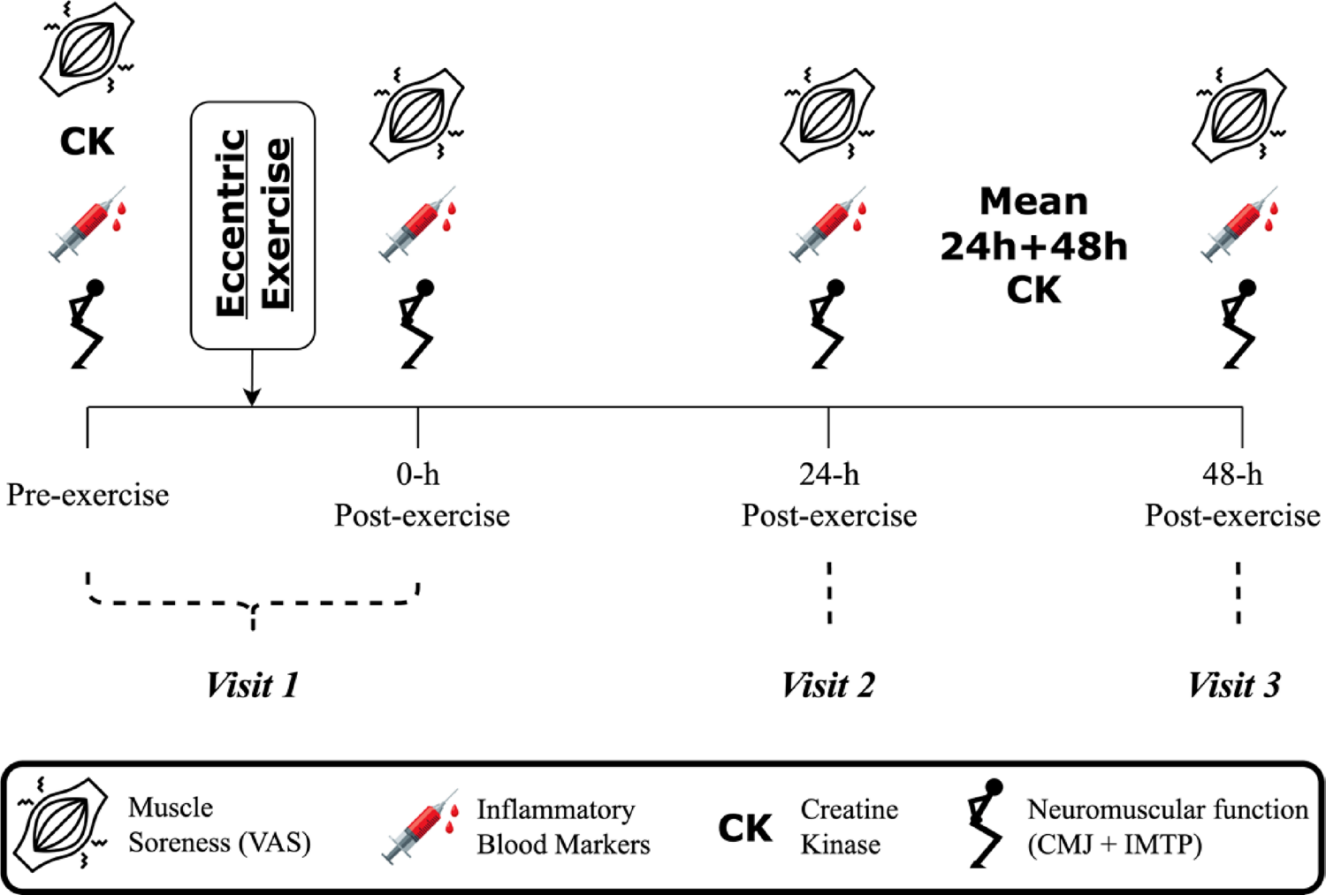
Eccentric muscle damage recovery visits. Participants completed 3 laboratory visits at week 0 and week 12

Participants completed 8 laboratory visits across 12 weeks, as previously described [32]. Visits 1–3 (week 0) and visits 6–8 (week 12) included physiological and biochemical assessments, peri-exercise and during recovery (Fig. 1). On visit 4 (week 4) and visit 5 (week 8) participants were assessed for anthropometry, whole blood fatty acids, physical activity and dietary intake [32]. Participants performed a lower-limb muscle damaging, eccentric cycling protocol at week 0 and 12. Perceived muscle soreness (VAS), neuromuscular function and inflammatory cytokines were assessed pre-exercise, 0-h, 24-h and 48-h post-exercise.

### Dietary supplementation

The DHA group received microencapsulated DHA via custom-formulated chewable tablets, while the Control group received sensory-equivalent tablets, with Sunola oil (from sunflower oil) as the primary fat source. Microencapsulation is increasingly being utilised to protect long-chain omega-3 fatty acids from oxidation and to improve stability in food matrices [33]. This delivery approach enables incorporation into chewable tablet formats or fortified foods, providing a practical ‘food-first’ approach for individuals not wanting to, or not able to swallow traditional fish oil capsules. Proprietary spray-dried carbohydrate-matrix microencapsulation technology was used to create a high oil-loading powder (either DHA or Sunola oil) before being compressed into a chewable tablet form (Nu-Mega Ingredients, Australia). Participants consumed a total of seven chewable tablets daily, spread across two meals, for 12 weeks. Control tablets provided 146 mg/d of omega-6 linoleic acid (LA; C18*:*2*n*-6) and 975 mg/d of monounsaturated oleic acid (OA; C18:1). The DHA tablets provided 715 mg/d DHA in triglyceride form. Full details of the dietary supplements are provided in Anthony et al. [32].

### Whole blood fatty acids and the Omega-3 Index

Whole blood fatty acids (24) were analysed and the O3I calculated at weeks 0, 4, 8 and 12 using dry blood spots collected from a finger-stick sample as detailed in Anthony et al. [32]. Participants ceased consumption of their tablets for 48-h prior to their week 12 sample. Samples were sent to an accredited laboratory (Fatty Acid Labs, Victoria, Australia) where each fatty acid in the whole blood was quantitatively identified using capillary column gas chromatography with standards and then described as a relative percentage of all twenty-four fatty acids (laboratory CV < 5%) [34]. The O3I was calculated by a validated regression equation (r = 0.96) using the sum of whole blood EPA + DHA [34].

### Eccentric muscle damage protocol

Concentric cycling peak power output (PPO) was established for each participant on an upright cycle ergometer (Wattbike Pro, Nottingham, UK). Participants were instructed to exert maximal effort for a 10-s sprint. Resistance (air) was manually increased during the 10-s bout to achieve a cadence of approximately 70–80 rpm whilst exerting maximal effort on the pedals. The peak power recorded during the 10-s bout was taken as the PPO. A target of 25–30% of PPO was used for the eccentric cycling protocol. To control for the eccentric muscle damage stimulus, week 0 absolute mean workload (W) during the eccentric intervals was maintained at the week 12 assessment.

After a 5-min rest period, participants were moved to a custom-built electric motor-driven semi-recumbent eccentric cycle ergometer. The eccentric cycling protocol consisted of 12 × 1-min intervals performed at 25–30% of PPO at 60 rpm with ~ 45-s rest separating each interval. A higher intensity, intermittent eccentric cycling protocol was selected in place of a lower intensity, steady state protocol as it has previously demonstrated a more pronounced effect on DOMS and perturbations in muscle damage markers [3]. Participants maintained their respective output Watts by modulating pressure applied to the pedals. Cadence remained at 60 rpm during each interval.

### Delayed onset muscle soreness assessment

Participants’ perceived muscle soreness was self-assessed using a 100 mm visual analogue scale (VAS), with 0-mm indicating “no pain” and 100-mm indicating “extreme pain”. Soreness ratings were recorded following the completion of a bodyweight squat (knee joint reaching ~ 90° degree knee flexion) at pre, 0-h, 24-h and 48-h post-exercise. Participants were asked to place a mark on the scale that corresponded with their perceived muscle soreness. To better capture only muscle pain (soreness), instruction was given to participants to try and distinguish pain from fatigue when completing their VAS assessment. Muscle soreness was quantified by manually measuring the completed VAS with a ruler to the nearest millimetre.

### Neuromuscular function assessments

Neuromuscular function was evaluated using countermovement jump (CMJ) and isometric mid-thigh pull (IMTP) assessments. These assessments were performed pre, 0-h, 24-h and 48-h post-exercise to quantify neuromuscular fatigue and recovery after the eccentric exercise bout at weeks 0 and 12. Participants began by completing the CMJ assessment, followed by the IMTP assessment, with a 5-min standing rest period between tasks.

#### Countermovement jump

Participants stood on force plates (Force Decks, VALD Performance, QLD, Australia) with feet centred, hands on hips, and eyes forward. After a 3-s countdown, they rapidly squatted to ~ 90° knee flexion and jumped without pausing, keeping legs straight during flight and landing in the same position. They performed three consecutive CMJ, with 60s rest between jumps. The best jump was used for analysis. Data were processed using VALD Hub performance software (VALD Performance, QLD, Australia).

#### Isometric mid-thigh pull

Participants first had their bar height set on a custom rig to achieve a hip angle of ~ 145° and a knee angle of ~ 125°, which was recorded and maintained for the week 12 assessments. Participants stood on force plates (Force Decks, VALD Performance, QLD, Australia) and gripped the bar with a double overhand grip, using weightlifting straps to prevent grip fatigue. After applying pre-tension and stabilising the force trace, they exerted maximal force for 5-s. The best of two IMTP pulls was used for analysis. Data were processed using VALD Hub performance software (VALD Performance, QLD, Australia).

### Biochemical indices of muscle damage and inflammation

#### Blood collection

Capillary blood (400–600 μl total whole blood) was collected via finger stick sampling into ethylenediaminetetraacetic acid (EDTA) coated microvettes (Sarstedt, Germany) at pre, 0-h, 24-h and 48-h post-exercise time points. The microvettes were sealed, mixed thoroughly by inversion and then centrifuged at 1000*g* for 10-min at 10 °C. The plasma layer (~ 150–250 μl) was collected, aliquoted and stored at – 80 °C for subsequent analysis of plasma creatine kinase and cytokine concentrations.

#### Creatine kinase

Plasma creatine kinase (CK) was measured in singlicate at pre-exercise and as a mean response to exercise over 48-h at week 0 and 12. The mean plasma CK response to exercise was determined by aliquoting 40 μl of plasma from the 24-h sample and 40 μl of plasma from the 48-h sample into a single Eppendorf tube. After thoroughly mixing the combined plasma (24-h + 48-h sample), 30 μl was pipetted onto reagent test strips and CK was quantified using spectrophotometry (Reflotron IV chemistry analyser). Prior to CK analysis, the spectrophotometer was calibrated using the manufacturer provided calibration strips (Reflotron Check). CK concentration is reported at units of enzyme activity per litre of blood plasma (U.L^−1^).

#### Inflammatory blood markers

Plasma cytokines were measured in duplicate pre, 0-h, 24-h and 48-h post-exercise at weeks 0 and 12. Plasma cytokine concentrations were analysed using a LEGENDplex multi-analyte bead-based immunoassay (BioLegend, San Diego, CA, USA), according to the manufacturer’s instructions. Data were acquired using an Accuri C6 Plus flow cytometer (BD Biosciences, Franklin Lakes, NJ, USA). This assay allowed simultaneous quantification of interleukin-6 (IL-6), tumour necrosis factor alpha (TNF-α), interleukin-10 (IL-10) and interleukin-1 receptor antagonist (IL-1RA).

Data were analysed using the LEGENDplex data analysis software suite (BioLegend). Replicate samples were averaged unless the coefficient of variation (CV%) was > 30%, in which case obvious outliers were removed or where possible, samples were rerun for confirmation.

### Standardisation and familiarisation

Participants were asked to limit any moderate to vigorous physical activity in the 24-h leading up to the muscle damaging protocol and for the next 48-h during their recovery visits. Participants were matched for weeks 0 and 12 time of day assessments to limit the influence of diurnal variation observed for plasma cytokines [35]. The mean time difference in the Control group was − 9 min [95% CI; − 26, 9] and in the DHA group it was − 6 min [95% CI; − 12, − 1]. Additionally, participants were balanced within group for AM/PM assessment time (Control: 50% AM (n = 6/12); DHA group: 58% AM (n = 15/26).

During their initial visit, participants were familiarised with eccentric cycling where they were provided demonstration and practice attempts. The ergometer was set to 60 rpm and verbal guidance was provided for when to apply pressure to the pedals. Power output was monitored via a Garmin Edge 530 connected to an SRM powercrank. Participants practiced maintaining ~ 10% of their PPO for 30-s and this was repeated with ~ 15% PPO after a 1-min rest. Familiarisation to the CMJ and IMTP assessments occurred at their first visit. Participants were given verbal instructions, demonstrations, and 3–5 practice attempts at 50–75% effort for the CMJ and IMTP tasks.

### Statistics

Data were analysed using Graphpad Prism software package (Version 10.2.1 for Mac, Graphpad Software, Boston, Massachusetts USA). Data were checked for normality with the Shapiro–Wilk test and skewed data were log-transformed prior to analysis. For outcome measures directly pertaining to the primary aims of the study, a two-way repeated measure analysis of variance (ANOVA) was used, with diet supplement (group: Control, DHA group) and time (week 0 and week 12, pre, 0-h, 24-h and 48-h post-exercise) main effects, and group x time interaction. Where a significant interaction or main effect was established, a post hoc Tukey test was conducted for comparisons of individual means. Week 0 variables, including anthropometry, physical activity levels and dietary intake were compared using an unpaired two-tailed t-test between Control and DHA groups.

Area under the curve (AUC) was calculated using the trapezoid rule for DOMS, inflammatory biomarkers, jump height and peak vertical force to represent the integrated response over 48-h.

The data collected are expressed as mean (95% CI). Log-transformed variables are presented as raw mean (95% CI). Alpha was set at *P* < 0.05. Cohen’s effect size (d) was calculated to compare magnitude of change between groups after supplementation for recovery metrics. Effect size values were classified as trivial (< 0.20), small (0.20–0.49), medium (0.50–0.79), and large (> 0.80) [36].

Two primary outcomes were prespecified; O3I and perceived muscle soreness. The estimated sample size for O3I (1:2 enrolment) was calculated at n = 7 Control and n = 14 DHA group participants (1.5% O3I difference, 1% SD, 80% power and 0.05 alpha). The estimated sample size for VAS measured perceived muscle soreness (1:2 enrolment) was calculated as n = 12 Control and n = 24 DHA group participants (30% difference in perceived soreness, 15mm SD, 80% power and 0.05 alpha). Therefore, the larger sample size requirement was chosen to ensure both outcomes were adequately powered. A 30% difference in perceived muscle soreness was selected based on similar studies demonstrating a 30% difference between groups, in addition to a minimal clinically significant difference in VAS-measure acute pain of approximately 30% where peak pain scores reach ~ 40–50mm.

## Results

### Participant characteristics, the Omega-3 Index and whole blood DHA

Participant characteristics for Control and DHA groups at week 0 are presented in Table 1. There were no differences between groups for anthropometry, physical activity, or dietary intake (*P* > 0.05).

**Table 1:** Participant characteristics at week 0 for Control and DHA groups

	Control		DHA		*P*-value
	Mean	95% CI	Mean	95% CI	
Age (y)	28	(23–32)	26	(24–28)	0.341
Weight (kg)	74.6	(64.5–84.6)	72.1	(67.1–77.0)	0.599
Height (cm)	174	(168–180)	176	(172–180)	0.676
Mod + Vig physical activity (mins/week)	417	(300–534)	433	(336–529)	0.844
Energy intake (kcal/day)	2219	(1809–2629)	2255	(2047–2464)	0.853
Protein intake (g/day)	116	(89–143)	113	(94–133)	0.880
Carbohydrate intake (g/day)	224	(177–272)	229	(205–252)	0.850
Fat intake (g/day)	83	(65–101)	90	(80–100)	0.423

Data analysed with a two-tailed unpaired t-test

Control (n = 12), DHA (n = 26)

Abbreviation Mod, Moderate; Vig, vigorous

The fatty acid results presented here are derived from inter-related data previously published in Table 3 of Anthony et al. [32] and are reproduced in text for clarity. There were no differences in whole blood DHA (Control: 2.26% [95% CI; 1.87, 2.65]; DHA group: 2.44% [95% CI; 2.17, 2.70], *P* = 0.411) or the O3I (Control: 4.75% [95% CI; 4.19, 5.30]; DHA group: 4.89% [95% CI; 4.53, 5.24], *P* = 0.622) between groups at week 0.

There was an interaction and main effect of group and time for whole blood DHA and the O3I (all *P* < 0.001). Whole blood DHA increased to week 8 in the DHA group, which was different to the Control group by that time point (Control 8wk: 2.11% [95% CI; 1.81, 2.42]; DHA group 8wk: 4.27% [95% CI; 4.01, 4.53], *P* < 0.001). The O3I increased to week 8 in the DHA group, which was different to the Control group by that time point (Control 8wk: 4.44% [95% CI; 4.01, 4.87]; DHA group 8wk: 7.32% [95% CI; 6.95, 7.68], *P* < 0.001). There was no further increase in either whole blood DHA or the O3I in the DHA group between week 8 and 12 (all *P* > 0.05), where at that final time point, both whole blood DHA (mean difference: 2.04% [95% CI; 1.64, 2.44], *P* < 0.001) and the O3I (mean difference: 2.60% [95% CI; 2.06, 3.14], *P* < 0.001) were elevated compared to the Control group. Comprehensive fatty acid data can be found in Anthony et al. [32].

### Concentric peak power output

Concentric peak power output (W) during the 10-s maximal effort sprint was not different between groups at week 0 (Control: 898W, [95% CI; 677, 1120]; DHA group: 957W, [95% CI; 831, 1082], *P* = 0.606).

### Eccentric protocol

The eccentric workload target of 25–30% of concentric peak power output (W) was achieved in both groups with no differences between the groups at week 0 (Control: 26.6%, [95% CI; 24.9, 28.2]; DHA group: 27.5%, [95% CI; 26.3, 28.7], *P* = 0.376). Following supplementation, both groups replicated the mean eccentric workload they achieved at week 0, with no interaction or main effects of group or time (Control 0wk: 235W, [95% CI; 183, 287], Control 12wk: 236W, [95% CI; 185, 286]; DHA group 0wk: 257W, [95% CI; 229, 285], DHA group 12wk: 258W, [95% CI; 230, 285], *P* > 0.05).

The efficacy of the eccentric cycling protocol to induce physiological perturbations was assessed at week 0 by combining participants from Control and DHA groups. Compared to pre-exercise values, the eccentric cycling protocol resulted in an increase in DOMS, along with a marked decrease in CMJ height and peak vertical isometric force at all time points (0-h, 24-h and 48-h post-exercise; all *P* < 0.01). Concentrations of the inflammatory cytokines IL-6 and TNF-α were elevated at all time points (0-h, 24-h, and 48-h post-exercise; all *P* < 0.01). IL-10 concentrations were elevated at 0-h and 48-h post-exercise (*P* < 0.05), while IL-1RA concentrations only increased at 48-h post-exercise (*P* < 0.05). The average CK response over 24-h and 48-h post-exercise was also higher compared to pre-exercise concentrations (*P* < 0.001).

### Delayed onset muscle soreness

**Fig. 2:**
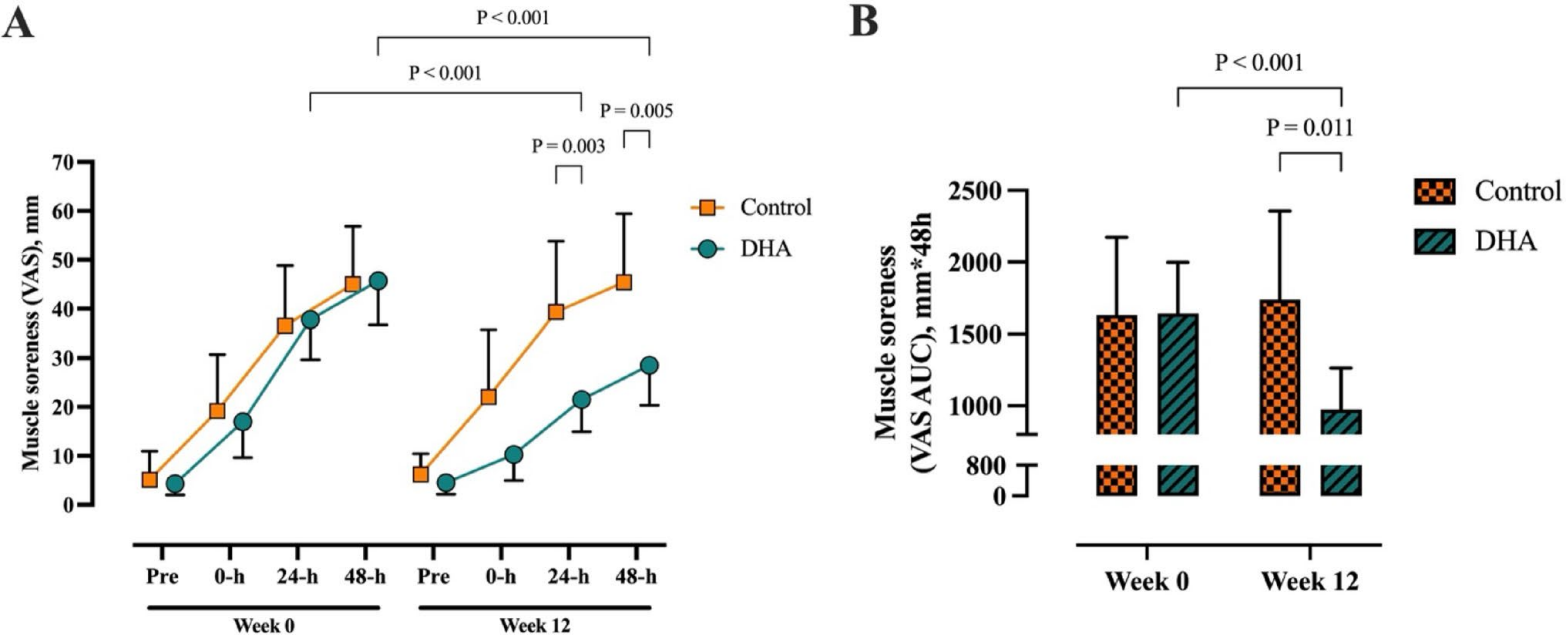
Perceived muscle soreness at week 0 and week 12 measured using a 100 mm VAS scale at pre, 0-h, 24-h and 48-h post-exercise. **A** Raw values measured at each time point. **B** AUC representing response over 48-h. Data presented as mean (95% CI). Control (n = 12), DHA (n = 26). Abbreviations: VAS, visual analogue scale

The 12-min eccentric cycling protocol resulted in an elevation in DOMS from pre-exercise at 24-h and 48-h time points in both Control and DHA groups (*P* < 0.001). Prior to supplementation, there were no differences in DOMS between groups at any time point (Fig. 2A). When the overall response was expressed as AUC over 48-h there remained no differences between Control and DHA groups at week 0 (Fig. 2B).

Following supplementation, there were no differences for DOMS in the Control group, compared to week 0, at any time point expressed as raw values (Fig. 2A) or the overall AUC response (Fig. 2B). There was an interaction and main effect of time (both *P* < 0.001) for DOMS, being lower in the DHA group and compared to the Control group by week 12 at 24-h and 48-h post-exercise (Fig. 2A). When expressed as AUC, there was a reduction in DOMS in the DHA group compared to week 0 and this was 56% lower compared to the Control group at week 12 (Fig. 2B). The between-group effect size at week 12 for AUC soreness was large (mean difference: 764 mm*48-h [95% CI; 99, 1428], Cohen’s *d* = 0.95).

### Neuromuscular function assessments

#### Countermovement jump (CMJ)

The performance and force–time metrics collected over 48-h from the CMJ assessment performed at weeks 0 and 12 are presented in Supplementary Table 1. Prior to supplementation and before exercise, there were no differences between the Control and DHA groups for jump height (Control: 32cm [95% CI; 27, 38]; DHA group: 32cm [95% CI; 29, 34], *P* = 0.746) or any other CMJ metric (Supplementary Table 1, *P* > 0.05). The 12-min eccentric cycling protocol resulted in a reduction in jump height from pre-exercise at the 24-h and 48-h post-exercise time points in both Control and DHA groups at week 0 (*P* < 0.001). When the jump height reduction over 48-h was expressed as AUC, there were no differences between Control and DHA groups at week 0 (Control: − 168 Δcm*48-h [95% CI; − 261, − 75]; DHA group: − 171 Δcm*48-h [95% CI; − 241, − 102], *P* = 0.941).

**Fig. 3:**
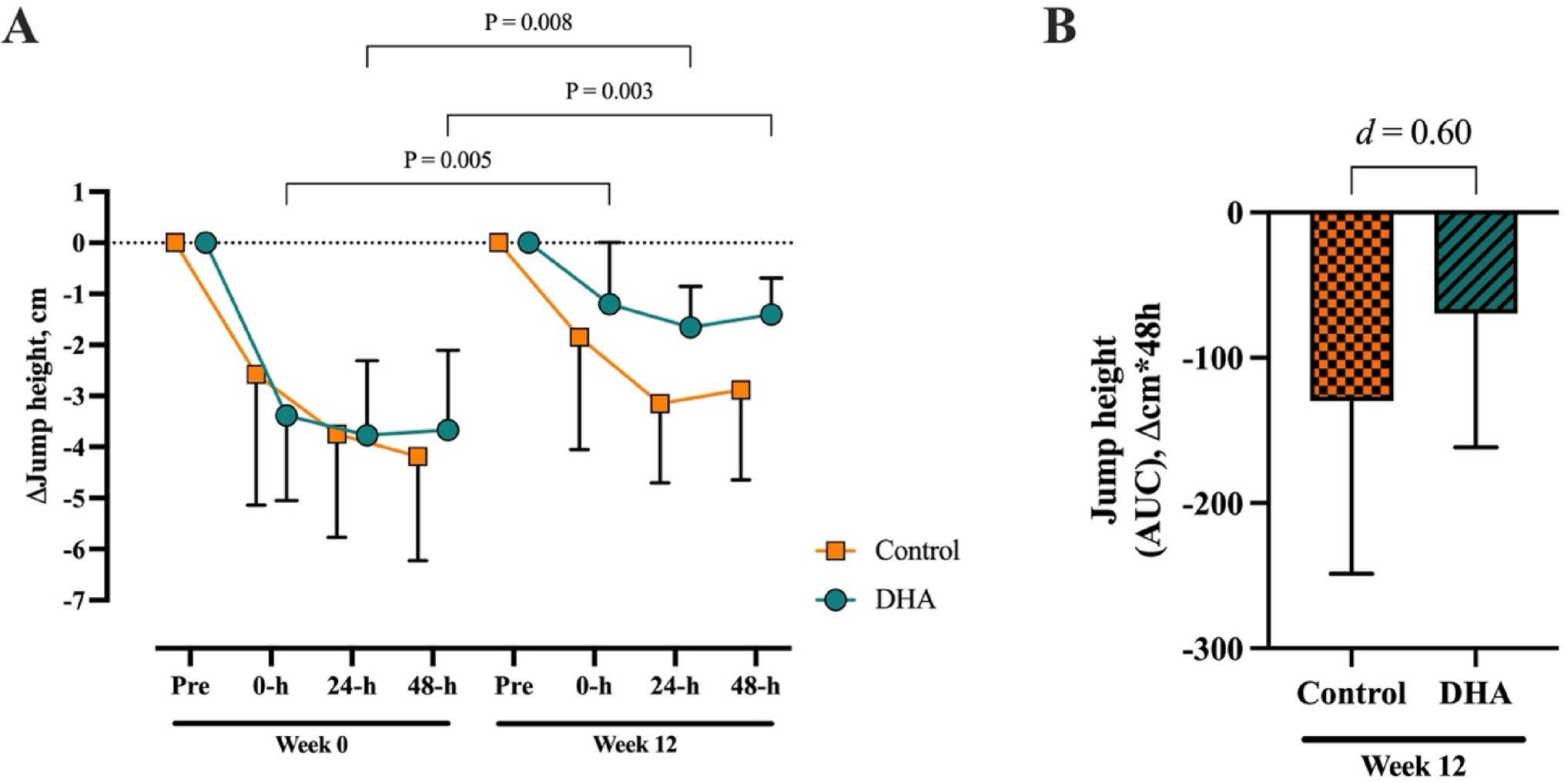
Change from pre-exercise for countermovement jump height at week 0 and week 12 measured **A** 0-h, 24-h and 48-h post-exercise and **B** as AUC representing the overall 48-h change in jump height from pre-exercise at week 12 with Cohen’s *d* effect size between groups. Data presented as mean (95% CI). Control (n = 12), DHA (n = 26).

There was no interaction or main effect of group for reduction in jump height, however there was a time effect (*P* < 0.001). Post hoc analysis indicated that the reduction in jump height observed following eccentric exercise was smaller at week 12 than at week 0 in the DHA group across the 0-h, 24-h, and 48-h time points (Fig. 3A, Supplementary Table 1). The between-group effect size at week 12 for AUC jump height was medium (mean difference: 60 Δcm*48-h [95% CI; − 21, 142], Fig. 3B).

There was no interaction or main effect of group for any CMJ metric, however all indices demonstrated a time effect, occurring in the DHA group at week 12 compared to week 0. Primarily, the components relating to the eccentric phase of the CMJ such as eccentric peak power and eccentric peak force were attenuated following DHA supplementation, particularly at the 48-h post-exercise time point (Supplementary Table 1).

#### Isometric mid-thigh pull (IMTP)

The performance and force–time metrics collected over 48-h from the IMTP assessment performed at weeks 0 and 12 are presented in Supplementary Table 2. Prior to supplementation and before exercise, there were no differences between the Control and DHA groups for peak vertical force (Control: 2399N [95% CI; 1820, 2978]; DHA group: 2410N [95% CI; 2079, 2741], *P* = 0.864) or any other IMTP metric (Supplementary Table 2, *P* > 0.05). When the peak vertical force reduction over 48-h post-exercise was expressed as AUC, there were no differences between Control and DHA groups at week 0 (Control: − 6312 ΔN*48-h [95% CI; − 9863, − 2760]; DHA group: − 6739 ΔN*48-h [95% CI; − 10,208, − 3270], *P* = 0.875).

**Fig. 4:**
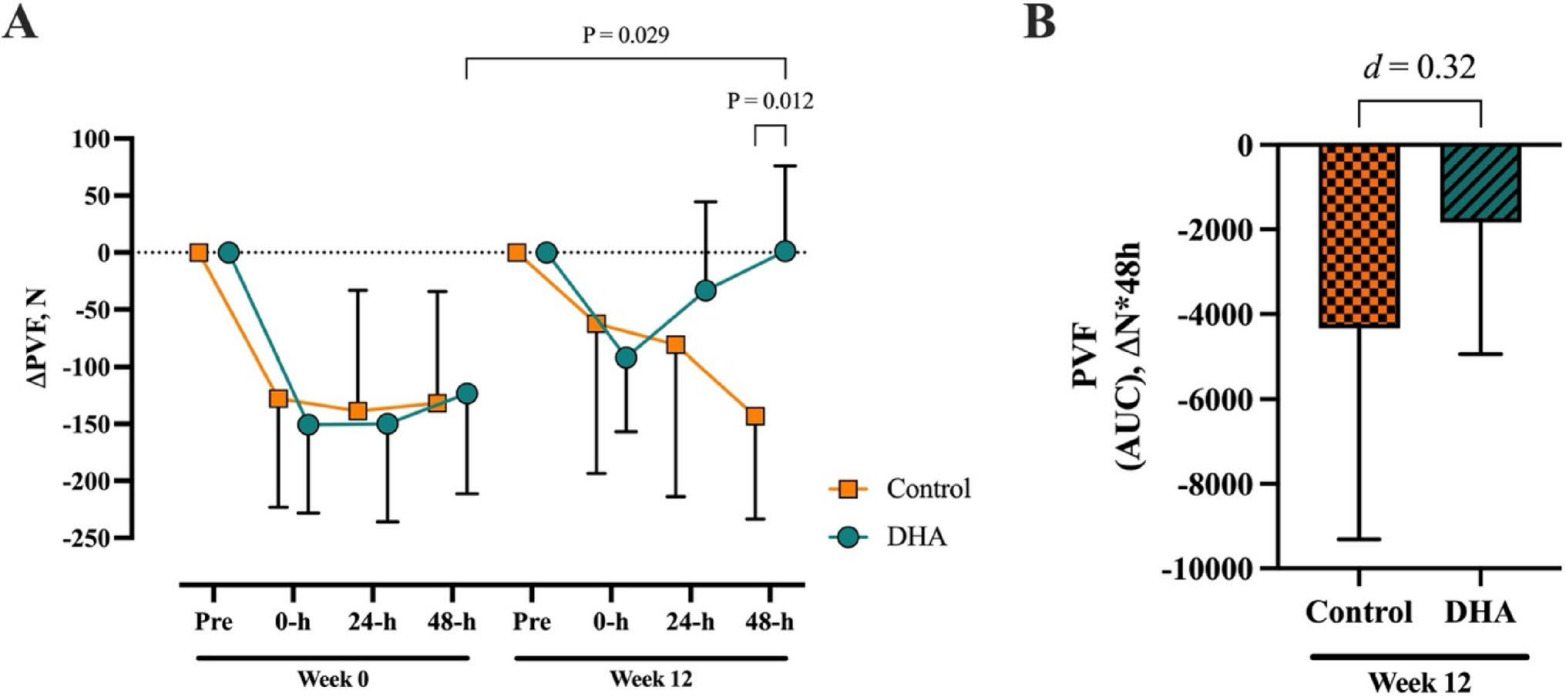
Change from pre-exercise for isometric mid-thigh pull PVF at week 0 and week 12 measured **A** 0-h, 24-h and 48-h post-exercise and **B** as AUC representing the overall 48-h change in PVF from pre-exercise at week 12 with Cohen’s *d* effect size between groups. Data presented as mean (95% CI). Control (n = 12), DHA (n = 26). Abbreviations: PVF, peak vertical force

There was no interaction or main effect of group for peak vertical force, however there was a time effect (*P* < 0.001). Post hoc analysis revealed that peak vertical force was improved in the DHA group by week 12 at 0-h, 24-h and 48-h post-exercise compared to week 0 (Supplementary Table 2). When peak vertical force was expressed as change from pre-exercise, there was no interaction or main effect of group, however there was a main effect of time (*P* < 0.001). Reduction in peak vertical force was attenuated at 48-h post-exercise in the DHA group by week 12 compared to week 0 and compared to the Control group (Fig. 4A). The between-group effect size at week 12 for AUC peak vertical force was small (mean difference: 2504 ΔN*48-h [95% CI; − 3149, 8159], Fig. 4B).

There were no interaction effects for any force–time metric, except for RFD 100-ms (Supplementary Table 2). All force–time metrics demonstrated a time effect, occurring in the DHA group at week 12 compared to week 0 (Supplementary Table 2). These occurred primarily within the first 100-ms time window.

### Biochemical indices of muscle damage and inflammation

#### Creatine kinase

CK values from two participants in the DHA group fell below the limit of detection, resulting in 12 Control and 24 DHA group participants with complete data at weeks 0 and 12. Pre-exercise CK was not different between the groups at week 0 or 12 (*P* > 0.05). There was no interaction or main effect of group for CK, however there was a main effect of time (*P* < 0.001). Post hoc analysis revealed that CK was higher post-exercise compared to pre-exercise at week 0 and week 12 in both the Control and DHA groups. Compared to pre-exercise values, and expressed as a percent change, CK was increased following eccentric exercise at weeks 0 and 12, with no differences between groups (Control 0wk: + 153% [95% CI; 5, 300]; DHA group 0wk: + 166% [95% CI; 61, 272], *P* = 0.893, Control 12wk: + 166% [95% CI; 0, 333]; DHA group 12wk: + 175% [95% CI; 30, 320], *P* = 0.932).

#### Inflammatory blood biomarkers

**Fig. 5:**
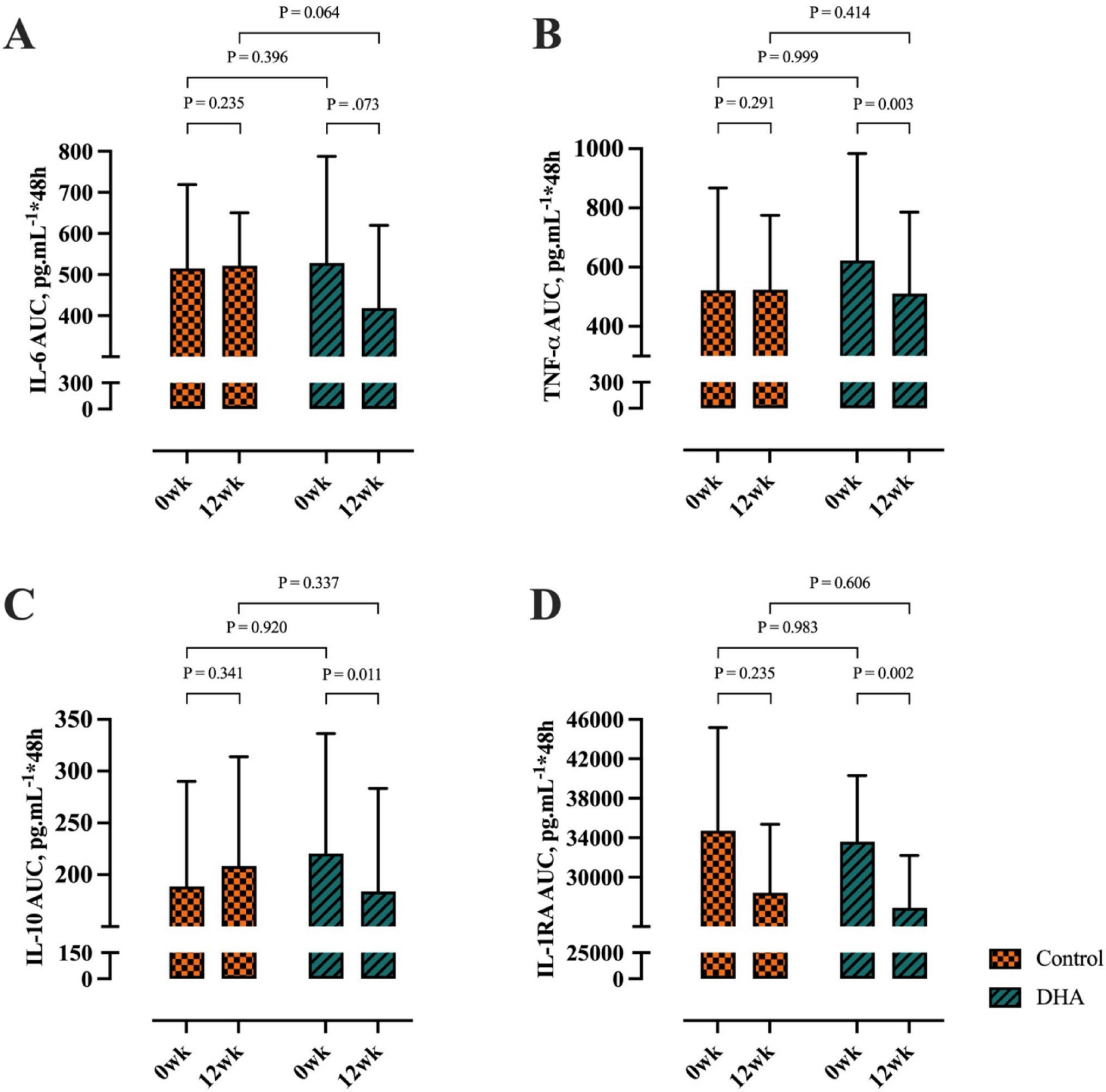
AUC of: pre-exercise; 0-h; 24-h; and 48-h post-exercise measures for **A** IL-6, **B** TNF-α, **C** IL-10, and **D** IL-1RA at week 0 and week 12. Data presented as mean (95% CI). Control (n = 12), DHA (n = 25). Abbreviations: IL-6, Interleukin-6; TNF-α, Tumour Necrosis Factor alpha; IL-10, Interleukin-10; IL-1RA, Interleukin-1 receptor antagonist

One participant was excluded from inflammatory cytokine analysis due to pre-exercise values falling below the limit of detection, leaving complete data for 12 Control and 25 DHA group participants over 48-h at weeks 0 and 12 (Supplementary Table 3). Cytokine concentrations were not different between groups at any time point prior to supplementation (Supplementary Table 3). When cytokine concentrations were expressed as 48-h post-exercise AUC, there were no differences between groups at week 0 for any cytokine (Fig. 5, *P* > 0.05). There was an interaction effect for the concentrations of TNF-α and IL-10 (Supplementary Table 3). There was no main effect of group for any cytokine concentration, however there was a main effect of time for all cytokine concentrations showing a general increase over 48-h post-exercise (Supplementary Table 3). When expressed as 48-h post-exercise AUC, there were interaction effects for IL-6 (*P* = 0.049), TNF-α (*P* = 0.010), and IL-10 (*P* = 0.026) and a main effect of time for IL-1RA (*P* = 0.007). Post hoc analysis revealed that TNF-α AUC and IL-10 AUC were lower in the DHA group at week 12 compared to week 0 (Fig. 5).

## Discussion

Addressing the key methodological limitations that have characterised previous LC n-3 PUFA studies in physically trained adults [37] and their application to augment recovery following eccentric exercise-induced muscle damage [11], the present study investigated whether a dietary achievable dose of DHA could enhance post-exercise recovery within a repeated measures design and using an eccentric cycling model. The findings demonstrated that DHA supplementation over three months achieved a substantial elevation in the O3I, with clear group separation by week 8, resulting in a clinically meaningful reduction in DOMS. Importantly, the eccentric cycling ergometer model effectively elicited marked physiological perturbations across 48-h which were reproducible after 12 weeks in the Control group.

### Delayed onset muscle soreness

Due to the repeated measures design, this is the first study which can comment on within group changes in DOMS. DOMS was equivalent between groups prior to supplementation and there was no alleviation of DOMS within the Control group following supplementation. Following three months of DHA supplementation, there was a reduction in DOMS within the DHA group at 24-h and 48-h post-exercise, and it was lower than the Control group. The minimum clinically significant difference in VAS-measured acute pain scores are estimated to be 9-mm [38]. For the current study, the within DHA group difference at 24-h and 48-h post-exercise for DOMS was 16-mm and 17-mm, respectively, highlighting a meaningful difference for participants engaged in regular physical training. Expressed as a percentage, the ~ 50% difference in DOMS between groups following supplementation is remarkably similar to other studies which have utilised a multi-joint eccentric stimulus in physically trained participants [24, 39], however the present study achieved this at an EPA + DHA dose between 3 and 11 fold lower. Visconti et al. [40], reported no differences between placebo and intervention groups despite using very high doses (≥ 6000 mg/d EPA + DHA) over 4 weeks. However, the validity of these findings are limited by the short supplement duration, absence of blood biomarker for confirmation of membrane incorporation, high inter-individual variability in DOMS and small sample sizes per group. Four studies have used a multi-joint eccentric stimulus in trained [24, 39, 40] and untrained [41] participants, yet none included more than 10 participants per intervention group. This raises the likelihood of a type II error, given that a sample size of at least ~ 14 intervention participants is needed to achieve 80% power for detecting a 30% difference in DOMS between groups.

A wide range of strategies have been investigated to alleviate DOMS, most of which are mechanical or thermal in nature, including massage, compression, cryotherapy, or heat therapy, and most often in exercise-naïve participants [42]. While these approaches may offer modest relief, their efficacy in physically trained individuals is limited. Pharmacological approaches, such as the use of NSAIDs, are also common among athletes [43] seeking to manage DOMS and accelerate return to training/game play, however their capacity to meaningfully reduce DOMS is not well supported [44]. NSAIDs primarily act through inhibition of COX, which can blunt the inflammatory response and potentially impair the adaptive processes essential for recovery [45]. In contrast, the findings of the present study support the efficacy of DHA consumption as a nutritional approach to attenuate DOMS, potentially acting through the promotion of inflammation resolution rather than suppression. These effects likely involve the upregulation of specialised pro-resolving mediators derived from DHA, which actively orchestrate the resolution of inflammation and tissue repair while maintaining the beneficial aspects of the inflammatory cascade [25]. This is particularly evident through the pronounced analgesic effects of specialised pro-resolving mediators in both animal and human models [46].

### Biochemical indices of muscle damage and inflammation

As a result of supplementation, no absolute concentration differences were observed in most cytokines at any time point, except for IL-6, which was lower at 48-h in the DHA group compared to the Control group at week 12. Blood levels of all the cytokines were higher after eccentric exercise. Modelling the 48-h cytokine response through AUC analysis demonstrated an attenuation in this post-exercise rise for TNF-α and IL-10 after supplementation in the DHA group, with IL-6 showing a similar but non-significant trend (*P* = 0.073). Importantly, this modulation of the inflammatory response was achieved following cessation of the dietary supplement for 48-h prior to the week 12 muscle damaging exercise. Whilst the current study did not measure metabolites involved in inflammation resolution, the results point towards pro-resolution effects of membrane incorporated DHA rather than a major reduction in the peak inflammatory response, as no such effect in peak cytokine concentrations were found. There is limited human data investigating blood levels of specialised pro-resolving mediators and changes occurring as a result of EPA + DHA supplementation, however Schaller et al. [47] has demonstrated that changes in the O3I are associated with increases in metabolites of resolution in patients with peripheral arterial disease. The ability of LC n-3 PUFA to modulate biomarkers of inflammation following EIMD has been variable across studies to date. It appears that the inflammatory modulating effects of LC n-3 PUFA are favoured in longer duration trials, or trials that demonstrate substantial elevations in blood levels of EPA and/or DHA [24, 48, 49], whereas these effects are not evident in trials lasting ≤ 4 weeks [50–53]. Therefore, sufficient time for membrane incorporation of EPA + DHA from supplementation is needed to observe changes in inflammatory profiles, likely mediated by specialised pro-resolving mediators. Most notably, the present study demonstrated alterations in inflammatory markers, underpinned by a meaningful elevation, and demonstrable plateau, in the O3I [32] confirming not only participant compliance but also membrane incorporation of DHA.

### Neuromuscular function

Countermovement jump and IMTP neuromuscular function deficits that commonly occur following eccentric cycling were attenuated in the DHA group, especially at the 48-h post-exercise time point. Specifically, DHA supplementation attenuated the decline in jump height and peak vertical force that was seen before supplementation and persisted in Control group. The greatest effect was seen in dynamic movement, as exhibited during CMJ, particularly in the force–time metrics associated with the eccentric or lowering phase of the jump. Equally, the rate of force development within the first 100-ms during the IMTP assessment was improved following DHA supplementation. This points towards a removal of inhibition to exert maximal effort rapidly, theoretically linked to a lower perception of DOMS. These effects of greater preservation of power and strength are in line with similar eccentric damage studies in trained [39] and untrained [14] participants using much higher EPA + DHA doses of approximately 4000 mg/d. In contrast, studies typically of shorter duration (≤ 4 weeks) generally do not report significant effects [40, 54, 55]. A recent study by Tsuchiya et al. [53] supplemented untrained participants for 4 weeks with 860 mg/d EPA + DHA and found no protective effects on muscular performance. Previous work from their laboratory using the same supplemental dose, yet at a longer supplement duration (8 weeks), did indeed find a beneficial effect on physical performance, including MVC torque [49, 56]. The time-dependent nature of DHA membrane incorporation suggests that supplementation duration can explain much of the variances observed between studies. Muscle membrane incorporation of EPA + DHA, even at doses exceeding those used in most eccentric damage studies, does not reach a plateau until after six weeks [57]. This is compounded by so few studies assessing a blood marker reflective of muscle membrane incorporation, such as erythrocyte EPA and/or DHA changes. While blood serum analysis of EPA + DHA is less representative of changes occurring in skeletal muscle membranes compared to erythrocyte membranes, it was reported in a series of studies by Tsuchiya et al. The null finding reported by Tsuchiya et al. [53] came with a ~ 30% increase in serum EPA + DHA, whereas they previously reported serum EPA + DHA increased by > 115% [56]. Notwithstanding, future emphasis should be placed on integrating the observed reduction in DOMS according to an increased O3I and explicit neuromuscular function.

## Limitations

Some limitations should be acknowledged when interpreting the findings of this study. First, although the sample size was appropriate for detecting the prespecified primary outcomes, it may have limited statistical power for some secondary measures such as inflammatory cytokines. Nevertheless, the total number of participants remains among the largest reported in trials investigating LC n-3 PUFA supplementation in recovery from exercise-induced muscle damage. Second, the eccentric cycling protocol was designed to ensure participants were truly naive to the motor pattern and to produce a consistent muscle damage stimulus. However, it must be acknowledged that it elicited only moderate levels of soreness, which may have constrained the detectable magnitude of between-group differences. Third, while the proposed role of specialised pro-resolving mediators in the resolution process provides a plausible mechanistic explanation for the reduction in DOMS, their quantification in human studies remains technically challenging [58]. Fourth, although erythrocyte incorporation of DHA serves as a robust biomarker of long-term intake and a surrogate for muscle membrane incorporation, future studies incorporating skeletal muscle biopsies would more directly confirm membrane incorporation [57] and strengthen mechanistic relevance. Finally, the present study is limited to the examination of post-exercise recovery over 48-h following an acute muscle damaging protocol. Extending the assessment of recovery to capture full physiological resolution, likely beyond 96-h, would provide a more comprehensive understanding of longer-term recovery.

## Conclusion

A dietary achievable dose of DHA, consumed over three months, effectively elevated the O3I, consistent with membrane incorporation of DHA. DHA supplementation attenuated DOMS and augmented physical performance recovery in trained male and female adults. Collectively, these findings demonstrate that increasing a previously low O3I through dietary DHA intake, in this case in the form of chewable tablets taken with a meal, can provide a practical approach to reduce muscle soreness and support recovery from eccentric muscle damaging exercise. Notably, these effects were not fully explained by the comparatively minor alterations in the inflammatory response, suggesting multi-factorial mechanisms beyond standard measures of inflammation.

## Supplementary Information

Below is the link to the electronic supplementary material.

Supplementary Material 1
