# Supplementary material for: Microencapsulated docosahexaenoic acid increases the Omega-3 Index and attenuates the physiological impact of eccentric exercise in physically trained adults: a 12-week double-blind placebo-controlled trial
Source: Eur J Nutr. 2026 May 29;65(4):147. doi: 10.1007/s00394-026-03998-6 (PMC13221426; doi:10.1007/s00394-026-03998-6)
Supplement: Supplementary file 1 — Supplementary Material 1 [file 394_2026_3998_MOESM1_ESM.docx]

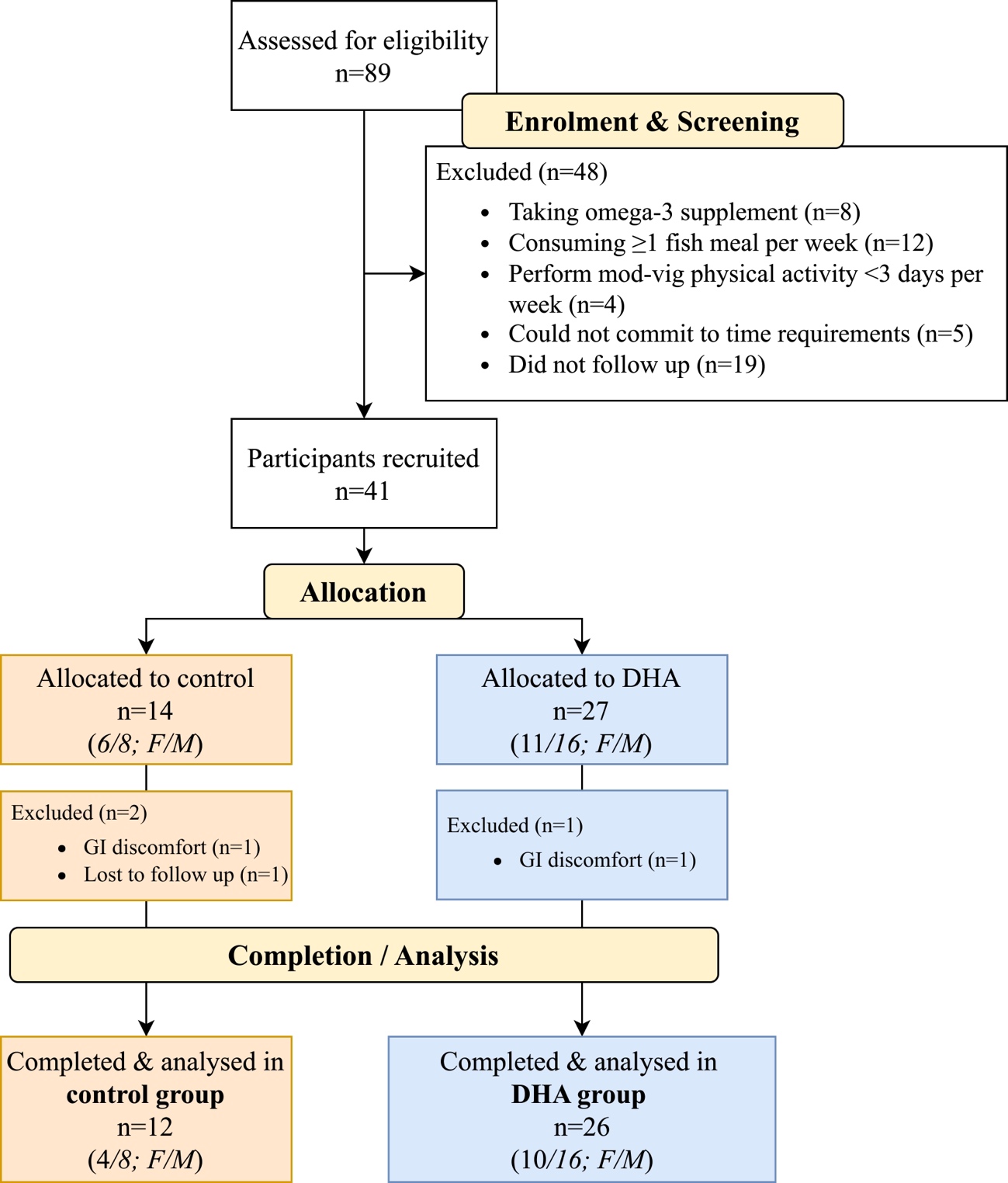


**Supplementary figure 1.** Participant screening, recruitment and completion flow chart. Adapted from Anthony et al. [32].

| **Supplementary table 1.** Countermovement jump performance and force-time metrics of control and DHA groups collected pre-exercise, 0-h, 24-h and 48-h post-exercise at week 0 and week 12 | | | | | | | | | | | |
| --- | --- | --- | --- | --- | --- | --- | --- | --- | --- | --- | --- |
|  | **Control 0Wk** | | **Control 12Wk** | | **DHA 0Wk** | | **DHA 12Wk** | | **P Value** | | |
|  | Mean | 95% CI | Mean | 95% CI | Mean | 95% CI | Mean | 95% CI | Time | Group | Time*Group |
| **Jump height (cm)** |  |  |  |  |  |  |  |  | <0.001 | 0.873 | 0.403 |
| Pre-exercise | 32.28 | (26.72-37.83) | 32.58 | (27.63-37.53) | 31.53 | (29.46-33.6) | 31.80 | (29.26-34.33) |  |  |  |
| Post-exercise | 29.70 | (24.26-35.14) | 30.73 | (25.74-35.72) | 28.15 | (25.88-30.41) | 30.60* | (28.36-32.83) |  |  |  |
| 24h post-exercise | 28.53 | (23.22-33.83) | 29.43 | (24.52-34.35) | 27.76 | (25.49-30.03) | 30.14* | (27.82-32.46) |  |  |  |
| 48h post-exercise | 28.09 | (22.80-33.38) | 29.70 | (24.45-34.95) | 27.87 | (25.53-30.20) | 30.39* | (28.00-32.79) |  |  |  |
| **Ecc peak power (W)** |  |  |  |  |  |  |  |  | <0.001 | 0.783 | 0.474 |
| Pre-exercise | 1185 | (917-1454) | 1373 | (1023-1724) | 1090 | (949-1231) | 1232 | (1088-1376) |  |  |  |
| Post-exercise | 995 | (731-1259) | 1261* | (921-1601) | 1037 | (898-1176) | 1240* | (1109-1371) |  |  |  |
| 24h post-exercise | 1066 | (772-1359) | 1180 | (875-1484) | 950 | (846-1053) | 1106* | (974-1237) |  |  |  |
| 48h post-exercise | 1038 | (654-1421) | 1148 | (841-1456) | 920 | (843-998) | 1144* | (1004-1284) |  |  |  |
| **Ecc peak force (N)** |  |  |  |  |  |  |  |  | <0.001 | 0.919 | 0.381 |
| Pre-exercise | 1614 | (1353-1874) | 1622 | (1324-1920) | 1558 | (1416-1700) | 1593 | (1456-1731) |  |  |  |
| Post-exercise | 1456 | (1225-1686) | 1567 | (1281-1853) | 1470 | (1344-1595) | 1597* | (1468-1725) |  |  |  |
| 24h post-exercise | 1473 | (1202-1744) | 1548 | (1257-1839) | 1419 | (1314-1525) | 1531* | (1408-1655) |  |  |  |
| 48h post-exercise | 1449 | (1160-1739) | 1537 | (1244-1830) | 1397 | (1293-1501) | 1562* | (1429-1696) |  |  |  |
| **Ecc mean deceleration force (N)** |  |  |  |  |  |  |  |  | <0.001 | 0.890 | 0.050 |
| Pre-exercise | 1224 | (1043-1406) | 1275 | (1042-1507) | 1176 | (1077-1276) | 1230 | (1132-1328) |  |  |  |
| Post-exercise | 1103 | (927-1279) | 1218* | (996-1441) | 1148 | (1050-1245) | 1246* | (1156-1335) |  |  |  |
| 24h post-exercise | 1134 | (935-1333) | 1184 | (965-1404) | 1100 | (1024-1175) | 1170* | (1086-1254) |  |  |  |
| 48h post-exercise | 1132 | (910-1354) | 1158 | (939-1376) | 1077 | (1009-1145) | 1194* | (1107-1282) |  |  |  |
| **Ecc peak power:con peak power** |  |  |  |  |  |  |  |  | <0.001 | 0.855 | 0.485 |
| Pre-exercise | 0.36 | (0.31-0.41) | 0.42 | (0.35-0.49) | 0.35 | (0.30-0.40) | 0.39 | (0.34-0.43) |  |  |  |
| Post-exercise | 0.32 | (0.25-0.39) | 0.38 | (0.30-0.46) | 0.34 | (0.29-0.39) | 0.39 | (0.35-0.43) |  |  |  |
| 24h post-exercise | 0.34 | (0.28-0.40) | 0.37 | (0.30-0.43) | 0.32 | (0.28-0.37) | 0.35 | (0.31-0.39) |  |  |  |
| 48h post-exercise | 0.32 | (0.23-0.42) | 0.35 | (0.28-0.42) | 0.31 | (0.27-0.35) | 0.36* | (0.31-0.40) |  |  |  |
| **Positive takeoff impulse (Ns)** |  |  |  |  |  |  |  |  | <0.001 | 0.761 | 0.926 |
| Pre-exercise | 283 | (230-337) | 294 | (240-349) | 271 | (246-295) | 279 | (253-306) |  |  |  |
| Post-exercise | 270 | (221-319) | 288 | (234-342) | 258 | (238-278) | 277* | (253-301) |  |  |  |
| 24h post-exercise | 271 | (219-323) | 282 | (229-335) | 254 | (234-274) | 271* | (246-296) |  |  |  |
| 48h post-exercise | 268 | (212-324) | 281 | (227-335) | 254 | (234-273) | 273* | (248-299) |  |  |  |
| **Concentric peak power (W)** |  |  |  |  |  |  |  |  | <0.001 | 0.973 | 0.827 |
| Pre-exercise | 3490 | (2728-4252) | 3420 | (2710-4130) | 3433 | (2941-3924) | 3437 | (3020-3854) |  |  |  |
| Post-exercise | 3359 | (2635-4083) | 3475 | (2731-4218) | 3222 | (2898-3545) | 3414 | (3020-3808) |  |  |  |
| 24h post-exercise | 3230 | (2577-3883) | 3333 | (2626-4040) | 3164 | (2822-3506) | 3376* | (2974-3778) |  |  |  |
| 48h post-exercise | 3360 | (2657-4062) | 3510 | (2636-4383) | 3218 | (2870-3566) | 3410 | (3004-3817) |  |  |  |
| **Concentric peak force (N)** |  |  |  |  |  |  |  |  | <0.001 | 0.836 | 0.855 |
| Pre-exercise | 1628 | (1371-1885) | 1657 | (1381-1933) | 1615 | (1470-1760) | 1651 | (1508-1794) |  |  |  |
| Post-exercise | 1553 | (1318-1789) | 1635 | (1356-1915) | 1581 | (1441-1721) | 1662* | (1505-1820) |  |  |  |
| 24h post-exercise | 1568 | (1323-1813) | 1629 | (1351-1907) | 1578 | (1440-1715) | 1659* | (1509-1809) |  |  |  |
| 48h post-exercise | 1587 | (1345-1828) | 1634 | (1371-1896) | 1598 | (1447-1748) | 1664* | (1511-1816) |  |  |  |
| Two-way repeated measures ANOVA with Tukey post-hoc multiple comparisons test  Control (n=12), DHA (n=26)  *Adjusted P<0.05 within group (week 0 vs week 12) | | | | | | | | | | | |

| **Supplementary table 2.** Isometric midthigh pull performance and force-time metrics of control and DHA groups collected pre-exercise, 0-h, 24-h and 48-h post-exercise at week 0 and week 12 | | | | | | | | | | | |
| --- | --- | --- | --- | --- | --- | --- | --- | --- | --- | --- | --- |
|  | **Control 0Wk** | | **Control 12Wk** | | **DHA 0Wk** | | **DHA 12Wk** | | **P Value** | | |
|  | Mean | 95% CI | Mean | 95% CI | Mean | 95% CI | Mean | 95% CI | Time | Group | Time*Group |
| **Peak Vertical Force (N)** |  |  |  |  |  |  |  |  | <0.001 | 0.823 | 0.285 |
| Pre-exercise | 2399 | (1820-2978) | 2506 | (1910-3103) | 2410 | (2079-2741) | 2470 | (2169-2772) |  |  |  |
| Post-exercise | 2271 | (1738-2805) | 2444 | (1869-3018) | 2259 | (1978-2540) | 2378* | (2111-2645) |  | | |
| 24h post-exercise | 2260 | (1757-2764) | 2426 | (1798-3053) | 2260 | (1977-2543) | 2437* | (2160-2713) |  | | |
| 48h post-exercise | 2267 | (1711-2824) | 2363 | (1808-2918) | 2286 | (2010-2563) | 2471* | (2187-2756) |  | | |
| **RFD 100ms (N/s)** |  |  |  |  |  |  |  |  | <0.001 | 0.706 | 0.047 |
| Pre-exercise | 4811 | (3887-5735) | 5278 | (4026-6530) | 4901 | (3892-5910) | 5193 | (4198-6187) |  |  |  |
| Post-exercise | 2967 | (1759-4175) | 3486 | (2497-4475) | 2824 | (1992-3656) | 4448* | (3398-5497) |  | | |
| 24h post-exercise | 2882 | (1752-4011) | 3275 | (1887-4662) | 2837 | (2058-3616) | 4172* | (3285-5059) |  | | |
| 48h post-exercise | 3005 | (1629-4381) | 3330 | (2160-4501) | 2998 | (2246-3750) | 4294* | (3570-5019) |  | | |
| **RFD 200ms (N/s)** |  |  |  |  |  |  |  |  | <0.001 | 0.952 | 0.408 |
| Pre-exercise | 4123 | (2877-5369) | 4093 | (2946-5239) | 4201 | (3433-4969) | 4085 | (3465-4705) |  |  |  |
| Post-exercise | 3390 | (2106-4673) | 3585 | (2457-4712) | 2930 | (2294-3566) | 3667* | (2939-4395) |  | | |
| 24h post-exercise | 3579 | (2332-4826) | 3415 | (2220-4611) | 3186 | (2482-3891) | 3336 | (2828-3845) |  |  |  |
| 48h post-exercise | 3127 | (1970-4284) | 3144 | (1993-4295) | 3007 | (2359-3655) | 3634 | (3008-4259) |  |  |  |
| **Force at 100ms (N)** |  |  |  |  |  |  |  |  | <0.001 | 0.941 | 0.519 |
| Pre-exercise | 1499 | (1283-1714) | 1604 | (1291-1916) | 1484 | (1286-1682) | 1562 | (1342-1782) |  |  |  |
| Post-exercise | 1257 | (1011-1504) | 1334 | (1102-1566) | 1278 | (1073-1483) | 1505* | (1270-1740) |  | | |
| 24h post-exercise | 1267 | (1000-1534) | 1385 | (1014-1756) | 1236 | (1076-1396) | 1460* | (1247-1673) |  | | |
| 48h post-exercise | 1362 | (941-1782) | 1369 | (1077-1660) | 1254 | (1096-1412) | 1408* | (1269-1547) |  | | |
| **Force at 200ms (N)** |  |  |  |  |  |  |  |  | <0.001 | 0.853 | 0.708 |
| Pre-exercise | 1804 | (1445-2164) | 1864 | (1505-2224) | 1766 | (1546-1985) | 1790 | (1576-2005) |  |  |  |
| Post-exercise | 1613 | (1248-1978) | 1704 | (1386-2022) | 1555 | (1350-1760) | 1738* | (1533-1942) |  | | |
| 24h post-exercise | 1637 | (1298-1976) | 1741 | (1340-2141) | 1561 | (1366-1756) | 1669 | (1483-1854) |  |  |  |
| 48h post-exercise | 1632 | (1232-2032) | 1663 | (1342-1983) | 1541 | (1350-1732) | 1693* | (1516-1869) |  | | |
| Two-way repeated measures ANOVA with Tukey post-hoc multiple comparisons test  Control (n=12), DHA (n=26)  *Adjusted P<0.05 within group (week 0 vs week 12) | | | | | | | | | | | |

| **Supplementary table 3.** Inflammatory blood markers (pg/mL) from control and DHA groups collected pre-exercise, 0-h, 24-h and 48-h post-exercise at week 0 and week 12 | | | | | | | | | | | |
| --- | --- | --- | --- | --- | --- | --- | --- | --- | --- | --- | --- |
|  | **Control 0Wk** | | **Control 12Wk** | | **DHA 0Wk** | | **DHA 12Wk** | | **P Value** | | |
|  | Mean | 95% CI | Mean | 95% CI | Mean | 95% CI | Mean | 95% CI | Time | Group | Time * Group |
| **IL-6** |  |  |  |  |  |  |  |  | <0.001 | 0.136 | 0.100 |
| Pre | 8.27 | (5.01-11.53) | 9.49 | (5.68-13.31) | 7.94 | (3.74-12.13) | 7.32 | (3.43-11.20) |  |  |  |
| Post | 11.75 | (6.83-16.67) | 11.68 | (8.01-15.35) | 9.40 | (5.10-13.70) | 9.17 | (5.05-13.29) |  |  |  |
| 24h post | 10.45 | (5.76-15.13) | 9.53 | (7.20-11.85) | 12.58 | (5.43-19.74) | 8.56 | (4.32-12.79) |  |  |  |
| 48h post | 10.33 | (5.85-14.80) | 12.74 | (8.90-16.58) | 9.66 | (4.85-14.47) | 8.63* | (4.29-12.97) |  |  |  |
| **TNF-⍺** |  |  |  |  |  |  |  |  | <0.001 | 0.777 | 0.003 |
| Pre | 8.09 | (2.99-13.19) | 8.97 | (3.35-14.59) | 10.88 | (4.93-16.82) | 10.48 | (4.75-16.20) |  |  |  |
| Post | 12.17 | (4.84-19.50) | 10.73 | (5.08-16.37) | 13.02 | (5.19-20.85) | 11.28 | (5.18-17.38) |  |  |  |
| 24h post | 10.27 | (2.87-17.67) | 10.22 | (6.10-14.33) | 12.86 | (5.10-20.63) | 10.29 | (4.73-15.84) |  |  |  |
| 48h post | 10.88 | (3.85-17.91) | 12.52 | (4.66-20.39) | 13.25 | (5.60-20.89) | 10.70 | (4.92-16.47) |  |  |  |
| **IL-10** |  |  |  |  |  |  |  |  | 0.043 | 0.598 | 0.015 |
| Pre | 3.34 | (1.61-5.06) | 3.69 | (1.66-5.72) | 3.87 | (1.77-5.97) | 3.56 | (1.64-5.48) |  |  |  |
| Post | 4.19 | (1.85-6.53) | 4.16 | (1.90-6.43) | 4.58 | (2.22-6.93) | 4.02 | (1.96-6.09) |  |  |  |
| 24h post | 3.75 | (1.58-5.92) | 4.01 | (2.08-5.95) | 4.56 | (1.98-7.14) | 3.65 | (1.61-5.68) |  |  |  |
| 48h post | 4.06 | (1.84-6.28) | 5.18 | (2.22-8.14) | 4.71 | (2.35-7.06) | 4.00 | (1.80-6.20) |  |  |  |
| **IL-1RA** |  |  |  |  |  |  |  |  | <0.001 | 0.901 | 0.806 |
| Pre | 574.67 | (365.42-783.92) | 465.53 | (263.79-667.27) | 632.47 | (485.32-779.62) | 521.08 | (390.88-651.29) |  |  |  |
| Post | 732.84 | (417.10-1048.57) | 562.59 | (328.61-796.56) | 598.59 | (442.29-754.89) | 489.07 | (358.21-619.92) |  |  |  |
| 24h post | 703.57 | (442.15-964.99) | 587.33 | (426.23-748.42) | 707.69 | (542.83-872.54) | 585.85 | (454.76-716.94) |  |  |  |
| 48h post | 759.76 | (498.93-1020.59) | 636.91 | (467.06-806.76) | 788.47 | (616.58-960.36) | 583.44 | (447.86-719.03) |  |  |  |
| Two-way repeated measures ANOVA with Tukey post-hoc multiple comparisons test  Control (n=12), DHA (n=25)  Abbreviations: IL-6, Interleukin-6; TNF-α, Tumour Necrosis Factor alpha; IL-10, Interleukin-10; IL-1RA, Interleukin-1 receptor antagonist  *Adjusted P<0.05 between groups | | | | | | | | | | | |
